# Biomedical Risk Factors of Achilles Tendinopathy in Physically Active People: a Systematic Review

**DOI:** 10.1186/s40798-017-0087-y

**Published:** 2017-05-18

**Authors:** Maria Kozlovskaia, Nicole Vlahovich, Kevin J. Ashton, David C. Hughes

**Affiliations:** 1 Faculty of Health Sciences and Medicine, Bond University, 14 University Drive, Robina, QLD 4226 Australia; 2 Department of Sports Medicine, Australian Institute of Sport, 1 Leverrier Street, Bruce, ACT 2617 Australia

**Keywords:** Achilles tendinopathy, Risk factors, Biomedical risk factors, Genetics

## Abstract

**Background:**

Achilles tendinopathy is the most prevalent tendon disorder in people engaged in running and jumping sports. Aetiology of Achilles tendinopathy is complex and requires comprehensive research of contributing risk factors. There is relatively little research focussing on potential biomedical risk factors for Achilles tendinopathy. The purpose of this systematic review is to identify studies and summarise current knowledge of biomedical risk factors of Achilles tendinopathy in physically active people.

**Methods:**

Research databases were searched for relevant articles followed by assessment in accordance with PRISMA statement and standards of Cochrane collaboration. Levels of evidence and quality assessment designation were implemented in accordance with OCEBM levels of evidence and Newcastle-Ottawa Quality Assessment Scale, respectively.

**Results:**

A systematic review of the literature identified 22 suitable articles. All included studies had moderate level of evidence (2b) with the Newcastle-Ottawa score varying between 6 and 9. The majority (17) investigated genetic polymorphisms involved in tendon structure and homeostasis and apoptosis and inflammation pathways.

Overweight as a risk factor of Achilles tendinopathy was described in five included studies that investigated non-genetic factors. *COL5A1* genetic variants were the most extensively studied, particularly in association with genetic variants in the genes involved in regulation of cell-matrix interaction in tendon and matrix homeostasis. It is important to investigate connections and pathways whose interactions might be disrupted and therefore alter collagen structure and lead to the development of pathology. Polymorphisms in genes involved in apoptosis and inflammation, and Achilles tendinopathy did not show strong association and, however, should be considered for further investigation.

**Conclusions:**

This systematic review suggests that biomedical risk factors are an important consideration in the future study of propensity to the development of Achilles tendinopathy. The presence of certain medical comorbidities and genetic markers should be considered when contemplating the aetiology of Achilles tendinopathy. Further elucidation of biomedical risk factors will aid in the understanding of tendon pathology and patient risk, thereby informing prevention and management strategies for Achilles tendinopathy.

**Trial Registration:**

PROSPERO CRD42016036558

## Key Points

- Increased BMI and adverse lipid profile may be important biomarkers of Achilles tendinopathy.
- Further research is required to confirm an association between genetic variation, of genes encoding collagen proteins and proteins involved in pathways of tendon homeostasis and Achilles tendinopathy.
- Genetic risk factors of Achilles tendinopathy may be modified by geographic factors.

## Background

Achilles tendinopathy is one of the most prevalent overuse tendon injuries associated with physical activities such as running and jumping [1]. It is the most common Achilles tendon disorder, with the highest incidence among runners, track and field athletes and volleyball, tennis and soccer players [1]. The term ‘tendinopathy’ is an umbrella term for the description of tendon conditions encompassing pain, swelling and impaired performance [2, 3]. Achilles tendinopathy can be acute or chronic. Diagnosis is usually made via a combination of clinical history, physical examination with or without medical imaging. The Victorian Institute of Sport Assessment-Achilles questionnaire (VISA-A questionnaire) may also be used to grade the severity of tendinopathy. In the acute phase, the cardinal symptoms are morning pain and stiffness and pain at the beginning and end of exercise sessions, with relief in between. The tendon is diffusely swollen, and there may be palpable crepitus. Tenderness is maximal 2–6 cm above the insertion. In chronic tendinopathy, the tendon remains painful with exercise but the tendon is nodular and thickened rather than swollen and oedematous [4, 5].

Originally, the pain of Achilles tendinopathy was attributed to an inflammatory process. While inflammatory cells have been observed, particularly in the early stages of Achilles tendinopathy, the response does not seem to be a traditional inflammatory response [6, 7]. Several models have been proposed to explain the aetiology of Achilles tendinopathy [8–10]. The continuum model of tendon pathology suggested by Cook and Purdam consists of three stages: reactive tendinopathy, tendon dysrepair and degenerative tendinopathy [9]. The first stage of the pathology results from acute overload of the Achilles tendon and can be characterised as a non-inflammatory proliferative response in the cell and matrix. The second stage is described as attempted tendon healing, through increased production of collagen and proteoglycans. Degenerative tendinopathy is the third stage and characterised by potentially irreversible changes in cell and matrix condition such as tenocyte apoptosis and matrix disorder [11]. Conservative, non-invasive management is the initial treatment of choice for Achilles tendinopathy, particularly in the early phases [4]. Recalcitrance to conservative management is not uncommon however, and some clinicians argue that conservative management of chronic Achilles tendinopathy is time consuming and unsatisfactory [5]. Surgery is sometimes recommended after exhaustion of conservative treatments, but response rates are variable [5].

Intrinsic and extrinsic aetiological factors interact in the genesis of Achilles tendinopathy. Intrinsic risk factors include demographic factors (sex, age, weight and height) and genetic polymorphisms; and local anatomical factors include leg length discrepancy, malalignment and decreased flexibility. Extrinsic factors comprise therapeutic agents (corticosteroids, antibiotics), environmental conditions, and physical activity-related factors, including training patterns, technique and equipment [1, 2]. The majority of the studies published over the last two decades are dedicated to the investigation of anatomical features and biomechanical faults as possible causes of Achilles tendinopathy. Several reviews have identified certain anatomic characteristics and biomechanical and training load errors as risk factors for Achilles tendinopathy [12–15].

The contribution of biomedical risk factors to the development of Achilles tendinopathy remains unclear. These may include medical comorbidities and physiological, biochemical and genetic factors. Imaging and histopathological analysis of the degenerative and recovery processes in tendon have provided a better understanding of the processes associated with tendon structure and metabolism. In terms of biochemical and metabolic processes, tendon tissue is relatively inert, with new collagen synthesis being a slow process [5]. Looking at the genetic coding of proteins comprising tendon structure may lead to a better understanding of the underlying molecular processes. Several genetic association studies have demonstrated that genetic polymorphisms influence collagen tissue structure, its turnover, degradation processes and therefore susceptibility to tendon injuries. Medical comorbidities and treatment of medical conditions also affect tendon structure and function [16, 17].

There is relatively little research focussing on potential biomedical risk factors for Achilles tendinopathy. The purpose of this systematic review is to identify studies and summarise current knowledge of biomedical risk factors of Achilles tendinopathy in physically active people. This information will contribute to the understanding of Achilles tendinopathy and may inform future prevention strategies.

## Methods

### Literature Search Strategy

This systematic review was registered in the international prospective register of systematic reviews PROSPERO (registration number CRD42016036558). Systematic review methodology included a thorough search of all accessible databases of scientific articles. Keywords were selected by following PICO (Patient, Intervention, Comparison, Outcome) principle [18]. Four databases (CINAHL, EMBASE, PubMed and SPORTDiscus) were searched using various combinations of the keywords: “risk OR predisposition OR genetic* OR aetiology OR etiology OR overuse OR pathogenesis” AND “Achilles OR Achilles Tendon” AND “tendinopathy* OR tendinitis OR tendonitis OR tendinosis OR injury OR pain” AND “exercise OR physical activity OR sport* OR run*”. Articles were assessed in accordance with the Preferred Reporting Items for Systematic Reviews and Meta-Analyses (PRISMA) statement [19] and the standards of Cochrane collaboration [18]. The search was completed in August 2016.

### Selection Criteria

Selected articles from each database were combined in one electronic library EndNote (Thomson Reuters, version X7). Duplicates were removed automatically in the electronic library. The titles and abstracts were then scanned, and clearly irrelevant studies and the remainder of the duplicates were removed. Following exclusion/inclusion criteria, two reviewers examined the abstracts of the remaining articles and selected studies for the full-text review. Inclusion criteria were the following: (a) retrospective and prospective studies, case-control studies and cohort studies investigating risk factors of Achilles tendinopathy; (b) clear definition of the studied injury and medical diagnosis of the injury; and (c) physically active people as studied subjects. Discrepancies in the lists of selected articles were resolved through the discussion between the reviewers.

### Quality Assessment

The quality of the included studies was evaluated applying the Newcastle-Ottawa Quality Assessment Scale (NOS) for case-control and cohort studies [20]. This assessment checklist is recommended by the Cochrane Handbook for Systematic Reviews of Interventions [18, 21]. The NOS checklist assesses quality of the articles in three domains: selection of the studied groups; comparability of the groups and control for confounding factors; and exposure for case-control studies or outcome for cohort studies. Total maximum score is nine for both types of studies. Results of the studies were critically analysed and presented in the narrative form reporting odds ratios (OR) and confidence intervals.

### Levels of Evidence

Oxford Centre for Evidence-based Medicine (OCEBM) levels of evidence was used as a guidance to evaluate selected articles. Levels of evidence could be identified according to the type of clinical question [22].

### Results Presentation

Results of the studies are described in narrative form, and odds ratios and confidence intervals or relative risks of developing Achilles tendinopathy are reported in Table 2 where possible.

## Results

**Fig. 1:**
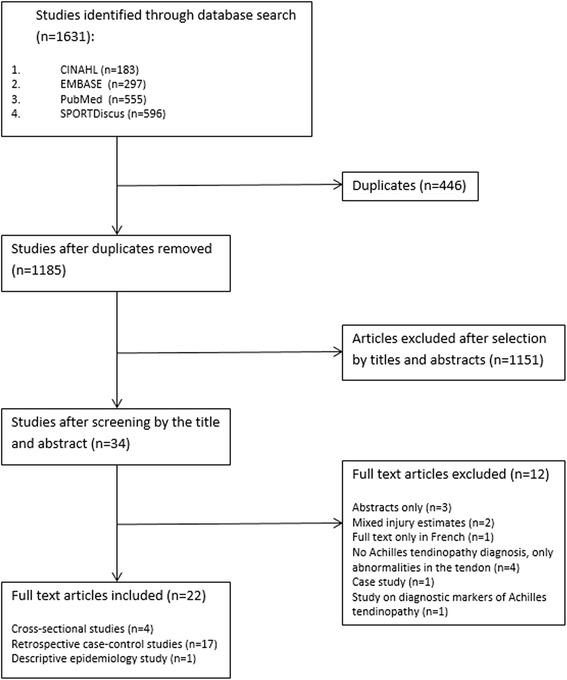
PRISMA flowchart of the study selection process

A total of 1631 articles were retrieved after the search of four databases. Following removal of duplicates, 1185 articles remained. Following screening of titles and abstracts, reviewers selected 30 articles as potentially relevant. The final 22 articles were included in the review after full-text screening and discussion between the reviewers. A flow chart of the article selection is given in Fig. 1. All included articles were published in English and had a mixed population of physically active people. Of the 22 included studies, 17 studies recruited runners and people who participated in recreational sports such as squash [23–39]. A single epidemiology study involved military personnel [40], while one study included elderly people involved in tennis, jogging and speed walking [41]. Another study recruited master and veteran athletes from race walking, long- and middle-distance running, jumping, hurdles and sprinting [42]. The remaining two studies recruited physically active people but did not identify the specific physical activities of the recruited cohorts [43, 44]. The majority of studies, 17 of 22, used the same definition of Achilles tendinopathy, assessing clinical diagnostic criteria of symptoms persisting in the Achilles tendon area [26]. One study’s clinical assessment included VISA-A questionnaire as an assessment tool [42]. Twenty-one out of 22 studies involved professional medical diagnosis of Achilles tendinopathy and additionally imaging records’ assessment. The epidemiology study examined over 80,000 medical records of military personnel relied on electronic medical record data [40].

The quality assessment using the NOS tool is presented in the Table 1. Three studies reached maximum score of nine [40, 42, 44], and the lowest score was six, shown in two studies [24, 28]. Studies with the score of nine included one epidemiologic cohort study of military personnel and two cross-sectional studies [40, 42, 44]. As the systematic review selection criteria excluded other systematic reviews, all included studies were assessed as moderate by the level of evidence (levels 3 and 4) (Table 2).

**Table 1:** The quality of the studies according to the Newcastle-Ottawa Quality Assessment Scale

References	Newcastle-Ottawa Quality Assessment Scale (NOS) score			Total score
	Selection	Comparability	Exposure/outcome	
Abate et al. (2015) [41]	3	2	3	8
Abrahams et al. (2013) [23]	4	1	2	7
Brown et al. (2016) [36]	4	1	2	7
El Khoury et al. (2016) [38]	4	2	2	8
El Khoury et al. (2015) [35]	4	2	2	8
El Khoury et al. (2013) [24]	4	1	1	6
Gaida et al. (2009) [43]	4	2	2	8
Gaida et al. (2010) [44]	4	2	3	9
Gibbon et al. (2016) [37]	4	2	2	8
Hay et al. (2013) [25]	4	1	2	7
Longo et al. (2009) [42]	4	2	3	9
Mokone et al. (2005) [26]	4	2	2	8
Mokone et al. (2006) [27]	4	2	2	8
Nell et al. (2012) [28]	4	0	2	6
Owens et al. (2013) [40]	4	2	3	9
Posthumus et al. (2010) [29]	4	2	2	8
Raleigh (2009) [31]	4	2	2	8
Rickaby et al. (2015) [39]	4	1	2	7
Saunders et al. (2013) [30]	4	2	2	8
September et al. (2008) [34]	4	2	2	8
September et al. (2009) [33]	4	1	2	7
September et al. (2011) [32]	4	1	2	7

**Table 2:** Summary of the included articles indicating biomedical risk factors of Achilles tendinopathy in physically active people

Author	Study design/level of evidence	Population characteristics						Injury definition	Identified risk factors
		Case/Control numbers	Age (year) (mean ± SD)	Sex (%)	BMI (kg/m^2^)	Not included (number)	Physical activity		
Abate et al. (2015) [41]	Cross-sectional study/3	38/38	CON, 69 ± 2.8; AT, 69.6 ± 3.3	CON and AT, 32 M and 6 F in each group	CON, 24.8 ± 2.3; AT, 26.8 ± 3	0	Speed walking, jogging, tennis	Pain at rest or during activities in the AT region, and/or local tenderness or swelling, and/or functional limitation (ankle dorsiflexion and extension)	Diabetes is a contributing factor to the development of AT (*p* = 0.004).
Abrahams et al. (2013) [23]	Retrospective case-control study/4	160 (81 SA + 79 AUS)/342 (149 SA + 93 AUS)	AT (age of initial injury), 39.8 ± 14.5; CON, 37.7 ± 11.7	AT, 73% M, 27% F; CON, 50.6%M, 49.4% F	AT 25.7 ± 3.8 (147) vs CON 24.2 ± 3.6 (330)	0	Long distance running, squash	As per Mokone et al. (2005)	*COL5A1* rs71746744, rs16399 and rs1134170 polymorphisms are independently associated with chronic AT (*p* = 0.008, OR = 2, 95%CI 1.2–3.3; *p* = 0.015, OR = 1.7, 95%CI 1.1–2.7; *p* = 0.014, OR = 1.8, 95%CI 1.1–2.9, respectively). *MIR608* rs4919510 polymorphism is associated with chronic AT (*p* = 0.023, OR = 1.6, 95%CI 1.1–2.5).
Brown et al. (2016) [36]	Retrospective case-control study/4	112 (87 AT and 25 RUP)/130 CON	CON, 41.6 ± 11.6 (123); ATP, 43.9 ± 13.8 (112)	CON, 63.1% M, 36.9% F; ATP, 60.7% M, 39.3% F	CON, 25.9 ± 4.5 (123); ATP, 26.0 ± 4.0 (82)	0	Not specified, physically active people	As per Mokone et al. (2005)	Three inferred allele combinations constructed from *COL5A1* rs12722, rs3196378 and rs71746744 increase risk of ATP, RUP and AT (*p* = 0.023, *p* = 0.011, *p* = 0.011). One inferred allele combination constructed from *CASP8* rs3834129 and rs1045485 was significantly associated with an increased risk of AT (*p* = 0.031).
El Khoury et al. (2016) [38]	Retrospective case-control study/4	118 (93 AT and 25 RUP)/130 CON	CON, 41.7 ± 11.6 (124); ATP, 43.7 ± 13.8 (117)	CON, 62.6% M, 37.4% F; ATP, 60.2% M, 39.8% F	CON, 25.9 ± 4.5 (123); ATP, 26.3 ± 4.1 (*n* = 86)	0	Not specified, physically active people	As per Mokone et al. (2005)	*TIMP2* rs4789932 variant is associated with ATP in males (*p* = 0.038). *MMP3* rs679620 variant is associated with RUP (*p* = 0.021).
El Khoury et al. (2015) [35]	Retrospective case-control study/4	135 (60 AUS + 75 SA)/239 (143 AUS + 96 SA)	CON, 38.2 ± 11.2 (230); AT, 40.1 ± 14.2 (129)	CON 50.6% M, 49.4% F; AT, 77.4% M, 22.6% F	CON 24.2 ± 3.6 (235); AT 25.7 ± 3.9 (124)	0	Running, high-impact sports	As per Mokone et al. (2005)	*FBN2* rs331079 variant is associated with ATP in males (*p* = 0.035). No association between *ELN* rs2071307 variant and ATP (*p* = 0.795).
El Khoury et al. (2013) [24]	Retrospective case-control study/4	165 (59 AUS + 114 SA)/248 (152 AUS + 96 SA)	AUS CON, 38.5 ± 11.9 (149); SA CON, 37.1 ± 10.0 (91); AUS ATP, 40.3 ± 14.1 (58); SA ATP, 40.2 ± 12.3 (107)	AUS CON, 39.7% M, 60.3% F; SA CON, 66.3% M, 33.7% F; AUS ATP, 67.8% M, 32.2% F; SA ATP, 73% M, 27% F	AUS CON 24.8 ± 4.0 (150); SA CON 23.3 ± 2.8 (93); AUS ATP 26.6 ± 4.1 (57); SA ATP 26.0 ± 3.9 (103)	20 Controls	Long distance running, squash	As per Mokone et al. (2005)	*TIMP2* rs4789932 variant is associated with ATP in combined SA and AUS populations (p = 0.016, OR = 1.58, 95% CI 1.03–2.43) None of the selected variants within the *ADAMTS2*, *ADAMTS5*, *ADAMTS14* and *ADAM12* genes were associated with risk of ATP in the two populations investigated (*p* = 0.316, *p* = 0.323, *p* = 0.849, *p* = 0.633, respectively).
Gaida et al. (2009) [43]	Cross-sectional study/3	60/60	CON, 47 ± 10; AT, 48 ± 9	CON, 53% M, 47% F; AT 53% M, 47% F	CON, 25 ± 3; AT, 25 ± 3	0	Not specified	Individuals with chronic Achilles tendon pain were diagnosed with midportion Achilles tendinopathy were included in the study.	AT subjects showed evidence of underlying dyslipidemia. They had higher triglyceride (TG), lower %HDL-C levels, apolipoprotein concentrations and higher TG/HDL-C ratio are associated with AT (*p* = 0.039, *p* = 0.016, *p* = 0.017, *p* = 0.036, respectively).
Gaida et al. (2010) [44]	Cross-sectional study/3	25/273	CON M 36.3 ± 11.3; CON F, 36.0 ± 10.3; AT M, 50.9 ± 10.4; AT F, 47.4 ± 10.0	CON, 40.3% M, 59.7% F; AT, 68% M, 32% F	CON M, 25.5 (3.5); CON F, 23.8 (3.2); AT M, 26.4 (3.2) AT F, 22.6 (2.6)	0	Not specified	Achilles tendons were examined by the ultrasound. Each tendon was classified as having a normal or abnormal internal structure. A tendon was classified as abnormal if any of the three following conditions were met: (1) one or more focal hypoechoic regions visible in both the longitudinal and transverse scans, (2) diffuse hypoechogenicity associated with bowing of the anterior tendon border or (3) diffuse hypoechogenicity associated with generalised thickening of the tendon in comparison with the contralateral tendon.	Older age and greater waist/hip ratio (WHR), higher android/gynoid fat mass ratio and higher upper-body/lower body fat mass ratio in men are associated with ATP (*p* < 0.001, 0.039, *p* = 0.014, *p* = 0.013, respectively). Men older than 40 years with a waist circumference >83 cm had the greatest prevalence of tendon pathology (33%). Older age, less total fat, less trunk fat, less android fat and lower central/peripheral fat mass ratio are associated with ATP in women (*p* = 0.008, *p* = 0.009, *p* = 0.003, *p* = 0.005, *p* = 0.004, respectively).
Gibbon et al. (2016) [37]	Retrospective case-control study/4	153 (99 AUS + 74 SA)/296 (199 AUS + 97 SA)	SA cohort from Mokone et al. (2005), AUS from Raleigh et al. (2009)	SA cohort from Mokone et al. (2005), AUS from Raleigh et al. (2009)	SA cohort from Mokone et al. (2005), AUS from Raleigh et al. (2009)	0	Not specified, physically active people	As per Mokone et al. (2005)	*MMP3* rs679620, rs3025058 inferred variants are associated with AT within the SA group (*p* = 0.012; OR 2.88; 95% CI 1.4 to 6.1).
Hay et al. (2013) [25]	Retrospective case-control study/4	184 (78 AUS + 106 SA)/338 (177 AUS +161 SA)	AUS CON, 39.4 ± 12.3 (174); SA CON, 36.4 ± 10.8 (154); AUS AT, 40.7 ± 14.5 (77); SA AT, 40.9 ± 14.8 (92)	AUS CON, 40.3% M, 59.7% F; SA CON, 63.8% M, 36.2 F ; AUS AT, 71.8% M, 28.2% F; SA AT, 67.6% M, 32.4% F	AUS CON 24.7 ± 3.9 (175); SA CON 23.6 ± 2.8 (151); AUS AT 26.2 ± 3.5 (75); SA AT 24.8 ± 3.3 (81)	0	Long distance running, squash	As per Mokone et al. (2005)	None of *COL11A1* rs3753841, rs1676486 and *COL11A2* rs1799907 variants are independently associated with AT (*p* = 0.108, *p* = 0.35, *p* = 0.154, respectively). TCT-inferred pseudohaplotype, constructed from the three studied polymorphisms is associated with an increased risk of AT (*p* = 0.006).
Longo et al. (2009) [42]	Cross-sectional study/3	85/93	CON, 52.4 ± 12.0; AT, 54.9 ± 11.8	No data	No data	0	Running, hurdle, jumping	VISA_A questionnaire was filled out by the participants in order to identify the presence of AT. If the score was less than 100, then they were examined by an orthopaedic surgeon to ascertain whether the AT diagnosis was appropriate.	Sex, age, weight, height and track and field specialty are not associated with AT (*p* = 0.14, *p* = 0.20, *p* = 0.21, *p* = 0.46, *p* = 0.032, respectively).
Mokone et al. (2005) [27]	Retrospective case-control study/4	114 (72 AT and 42 RUP)/127	CON, 40.4 ± 10.8 (120); ATI, 39.8 ± 13.3 (112)	CON, 63.5% M, 36.5% F; ATI, 72.8% M, 27.2% F	CON, 23.3 ± 2.7 (120); ATI, 26.0 ± 4.0 (112)	0	Running, squash	The clinical diagnostic criteria for chronic Achilles tendinopathy were gradual progressive pain over the posterior lower limb in the Achilles tendon area for greater than 6 months, together with at least one out of the following six criteria: (1) early morning pain over the Achilles tendon area, (2) early morning stiffness over the Achilles tendon area, (3) a history of swelling over the Achilles tendon area, (4) tenderness to palpation over the Achilles tendon, (5) palpable nodular thickening over the affected Achilles or (6) movement of the painful area in the Achilles tendon with plantar-dorsi-flexion (positive “shift” test).	*TNC* 12 and 14 GT repeats variants frequencies were significantly higher in the symptomatic subjects, whereas the frequencies of 13 and 17 GT repeats variants were significantly higher in the asymptomatic control subjects (*p* = 0.001). 13 or 17 GT repeats were associated with 6.2 times lower risk of AT (OR = 6.2, 95%CI 3.5–11, *p* < 0.001).
Mokone et al. (2006) [27]	Retrospective case-control study/4	111 (72 AT + 39 RUP)/129	CON, 40.3 ± 11.0 (122); ATP, 40.1 ± 14.0 (108); AT, 39.7 ± 15.3 (69); RUP, 40.8 ± 11.3 (39)	CON, 61.7% M, 28.3% F; ATP, 73% M, 27% F; AT, 73.5 M, 26.5% F; RUP 79.5% M, 20.5% F	CON, 23.2 ± 2.7 (121); ATP, 25.9 ± 3.9 (108); AT, 24.7 ± 3.3 (69); RUP, 28.1 ± 4.1(39)	0	Running, recreational sports	As per Mokone et al. (2005)	Three alleles produced by the BstUI RFLP within the 3′-UTR of the *COL5A1* alleles produced by BstUI RFLP within the 3′-UTR are associated with ATP and AT (*p* = 0.006, *p* = 0.0009, respectively).
Nell et al. (2012) [28]	Retrospective case-control study/4	166 (87 SA + 79 AUS)/358 (159 SA + 199 AUS)	No data	No data	No data	0	Running, recreational sports	As per Mokone et al. (2005)	*CASP8* rs3834129 variant is associated with AT (*p* = 0.0294, OR = 1.67; 95% CI 1.08–2.60)
Owens et al. (2013) [40]	Descriptive epidemiology study/3	450/77,092	Cohort was divided into groups by year born; mean age was not calculated.	CON, 70.3% M, 29.7% F; AT, 69.33% M, 30.67% F	CON, underweight/normal 44.76%, overweight 46.54%, obese 8.7%; AT, underweight/normal 35.11%, overweight 51.33%, obese 13.56%	0	Military training	International Classification of Diseases, 9th Revision (ICD-9) represents tendinopathies that may have been caused by acute injury or the result of chronic pathology.	Overweight, obesity and moderate alcohol consumption are associated with AT (AOR = 1.29, 95% CI 1.04–1.59; AOR = 1.59, 95% CI 1.16–2.17; AOR = 1.33, 95% CI 1–1.76, respectively).
Posthumus et al. (2010) [29]	Retrospective case-control study/4	171 (59 AUS + 73 SA)/235 (142 AUS + 96 SA)	AUS CON, 39.0 ± 12.1 (140); AUS ATP, 40.3 ± 14.1 (59); SA CON, 36.9 ± 9.9 (89); SA ATP, 40.2 ± 13.5 (107)	AUS CON, 40.2% M, 59.8% F; AUS ATP, 67.8% M, 32.2% F; SA CON, 66.3% M, 33.7% F; SA ATP, 73% M, 27% F	AUS CON, 24.9 ± 4.0 (141); AUS ATP, 26.6 ± 4.1 (57); SA CON, 23.3 ± 2.8 (93); SA ATP, 26.0 (103)	0	Running, recreational sports	As per Mokone et al. (2005)	*GDF5* rs143383 variant is associated with AT in AUS population and combined AUS and SA populations (*p* = 0.011, OR = 2.24, 95%CI 1.21–4.16; *p* = 0.004, OR = 1.82, 95%CI 1.23–2.74, respectively). No association between the functional *TGFB1* rs1800469 variant and ATP (*p* = 0.491).
Raleigh et al. (2009) [31]	Retrospective case-control study/4	114 (75 AT and 39 RUP)/ 98	CON, 36.8 ± 9.9 (91); AT, 40.5 ± 13.7 (70); RUP, 40.7 ± 11.5 (37)	CON, 67% M, 33% F; AT, 73% M, 27% F; RUP 73% M, 27% F	CON, 23.3 ± 2.8 (95); AT, 24.9 ± 3.4 (66); RUP, 27.8 ± 3.7 (37)	0	Not specified, physically active people	As per Mokone et al. (2005)	*MMP3* rs679620, rs591058 and rs650108 are associated with AT (*p* = 0.010, OR = 2.5, 95% CI 1.2–4.9; *p* = 0.023, OR = 2.3, 95% CI 1.1–4.5; *p* = 0.043, OR = 4.9, 95% CI 1.0–24.1, respectively). An inferred haplotype of these three SNPs is significantly under-represented in AT cases and may be protective against the development of AT (*p* = 0.038).
Rickaby et al. (2015) [39]	Retrospective case-control study/4	131/131	CON, 41.3 ± 11.3 (122); ATP, 54.1 ± 14.2 (127)	CON, 62.6% M, 37.4% F; ATP, 61.8% M, 38.1% F	CON, 25.7 ± 5.1 (122); ATP 26.3 ± 4.1 (94)	0	Not specified, physically active people	As per Mokone et al. (2005)	*TNFRSF1A* rs4149577 and *CASP8* rs3834129 SNPs were not associated with ATP (*p* = 0.335, *p* = 0.635, respectively).
Saunders et al. (2013) [30]	Retrospective case-control study/4	179/339	No data	No data	No data	0	Running, recreational sports	As per Mokone et al. (2005)	A haplotype of *COL27A1* rs946053, *TNC* rs13321 and rs2104772 variants is significantly associated with risk of AT in a SA and AUS cohorts (*p* = 0.019).
September et al. (2008) [34]	Retrospective case-control study/4	137 (93 AT and 44 RUP)/131	CON, 37.1 ± 10.4 (124); ATI 40.0 ± 13.5 (131); AT, 39.1 ± 14.3 (87); RUP, 41.8 ± 11.6 (44)	CON, 64.6 M, 35.4 F; ATI, 73.7% M, 26.3 F; AT, 72% M, 28% F; RUP, 77.3% M, 26.6% F	CON, 23.3 ± 2.7 (126); ATI, 25.8 ± 3.8 (128); AT, 24.8 ± 3.3 (84); RUP, 27.8 ± 4.0 (44)	0	Running, recreational sports	As per Mokone et al. (2005)	No association between *COL12A1* rs240736, *COL14A1* rs4870723 and AT (*p* = 0.992, *p* = 0.232, respectively).
September et al. (2009) [33]	Retrospective case-control study/4	178 (85 AUS + 93 SA)/342 (210 AUS + 132 SA)	AUS CON, 38.5 ± 12.4 (205); AUS AT, 40.4 ± 14.2 (84); SA CON and AT are the same as in Mokone et al. (2006)	AUS CON, 40.2% M, 59.8% F; AUS AT, 72.9% M, 27.1% F	AUS CON, 24.6 ± 3.9 (207); AUS AT, 26.5 ± SD 3.8 (82)	0	Running, recreational sports	As per Mokone et al. (2005)	*COL5A1* rs12722 variant is associated with AT in AUS cohort (*p* = 0.001). *COL5A1* rs12722 variant had a significantly decreased risk of AT in AUS and SA cohorts (*p* = 0.017, OR 0.42, 95% CI 0.20–0.86; *p* = 0.008, OR 0.38, 95% CI 0.18–0.77, respectively). *COL5A1* rs13946 is not associated with AT (*p* = 0.197).
September et al. (2011) [32]	Retrospective case-control study/4	175 (90 SA + 85 AUS)/369 (161 SA + 208 AUS)	No data	No data	No data	0	Running, recreational sports	As per Mokone et al. (2005)	No association of *IL1β* (two polymorphisms), *IL6* or *IL1RN* genes and AT (*p* = 0.380, *p* = 0.097, *p* = 0.183, *p* = 0.779, respectively). The inferred allele combination constructed from the *COL5A1* BstUI RFLP – *IL1β* −31 T → C – *IL1β* −511C → T – *IL6* − 172G → C – *IL1RN* VNTR is associated with AT (*p* = 066005) and suggests the potential interactions of these respective sequence variants in modulating the risk profile of developing AT.

*Abbreviations*: *AT* Achilles tendinopathy, *BMI* body mass index, *F* female, *M* male, *SA* South African, *AUS* Australian, *RUP* Achilles tendon rupture, *ATP* Achilles tendon pathology, *ATI* Achilles tendon injury, *CON* uninjured control, *HDL-C* high-density lipoproteins-cholesterol, *RFLP* restriction fragment length polymorphism, *VNTR* variable number tandem repeat

## Discussion

The selected studies focused on a diverse range of biomedical risk factors for Achilles tendinopathy such as demographic factors, chronic medical conditions, lifestyle habits and genetic factors, including those contributing to collagen structure and tendon homeostasis and those involved in apoptosis and inflammation. The majority of the included studies were case-control studies (Fig. 1). The main limitation of the included studies was their level of evidence. According to the recommended OCEBM hierarchy, cohort and case-control studies approach levels of evidence 3 and 4, with systematic reviews and randomised controlled trials providing levels 1 and 2 evidence [45].This limitation must be taken into consideration when reviewing the results of these studies. Here, we discuss the different approaches undertaken and highlight the main findings obtained in the search for the key biomedical risk factors for Achilles tendinopathy.

### Non-Genetic Biomedical Risk Factors

Biomedical risk factors include non-modifiable factors such as age, sex, weight and chronic medical conditions. A cross-sectional study on 178 master track and field athletes did not find any association between Achilles tendinopathy (as determined by VISA-A scores) and age, sex, weight or height [42]. However, the recruitment approach used in this study may have resulted in unintentional exclusion of those with the most severe AT as these athletes may not have been able to compete at the events where recruitment took place.

A study of 450 cases of Achilles tendinopathy in a large military personnel cohort showed that overweight and obesity had a strong association with the development of the injury. Additionally, this study found that moderate alcohol use was associated with increased risk of Achilles tendinopathy. However, the magnitude of the OR was modest and could be explained by the possible influence of alcohol on metabolic and inflammatory factors [40]. A case-control study of 60 patients with mid-portion Achilles tendinopathy and 60 matched uninjured controls by age, sex and body mass index (BMI) showed that patients with Achilles tendinopathy had higher levels of triglycerides (TG), lower levels of high-density lipoprotein cholesterol (HDL-C) and higher TG:HDL-C ratio. These lipid profiles are typical for insulin resistance syndrome and usually described as dyslipidemia. This finding suggests that serum lipids may be involved in the development of Achilles tendinopathy [43, 44]. A second study investigated fat distribution on male and female patients with asymptomatic Achilles tendinopathy. Ultrasound examinations of Achilles tendon in 298 participants identified a higher pathology rate among men than women (17/127, 13%, versus 8/171, 5%). Males with asymptomatic Achilles tendon pathology had elevated waist:hip ratio, were older and had higher central/peripheral fat mass and larger waist circumferences (above 83 cm) compared to individuals without tendon pathology. This pattern of fat tissue distribution around the abdominal area is usually associated with metabolic syndrome and might be linked to the previously reported association between dyslipidemia and Achilles tendinopathy. Surprisingly, women with asymptomatic Achilles tendon pathology had lower central:peripheral fat mass ratios compared to women without pathology, which could be explained by the effect of oestrogen on body fat distribution, a factor that was not investigated in this study [44]. A recent study comparing patients with Achilles tendinopathy aged over 65 years old to matching uninjured controls showed significantly higher prevalence of diabetes, higher BMI and higher level of sport participation in the injured group [41]. These findings are consistent with other studies which indicate susceptibility in those with chronic comorbidities. The types of exercises undertaken by the subjects (speed walking, jogging and tennis) were relatively high risk in nature and may have been beyond the loading capacity of the tendon in ageing individuals [41]. The link between adverse lipid profile and tendinopathy could be explained by adipokine modulation of certain enzymes’ production which are important for tenocyte functioning. Additionally, chronic low-grade inflammation, which is typical for obesity, may affect tendon healing process. At the same time, tendon healing is also disrupted by low concentrations of immune cells that tend to migrate into adipose tissue [46]. A systematic review of 17 articles on the link between lipid profile and tendon health showed a strong association between tendon pathology and high lipid parameters [47].

### Genetic Contributors to Collagen Structure and Tendon Homeostasis

Genetic variations are alterations in DNA sequence among individuals that may account for differences in phenotype and occasionally in health state. Genetic variations occurring in more than 1% of a population are considered useful polymorphisms for genetic linkage analysis. One of the most commonly investigated types of genetic variation is the single-nucleotide polymorphism (SNP), a variation in a single nucleotide located at a specific position in the DNA [48]. The genetic studies included in this review all employed a candidate gene approach in order to find genetic polymorphisms in genes that might influence tendon structure and its development and therefore either predispose to or protect from the development of Achilles tendinopathy. Genetic predisposition has long been proposed as a contributor to the development of Achilles tendinopathy. Early studies assessed the genetic association between ABO blood type and tendon injuries, described in several investigations of Finnish and Hungarian patients [49, 50]. However, further research did not support this link, but identified the chromosomal region of interest on the long arm of chromosome 9 (9q), which carries genes closely linked to the ABO blood group gene [27].

This review includes 14 studies investigating 16 genes linked to collagen structure and tendon homeostasis. Two of these investigated genes located in 9q region, particularly tenascin-C (*TNC*) and *COL5A1* [26, 27]. Both studies found an association of polymorphic variants of *TNC* and *COL5A1* genes with the development of Achilles tendinopathy. Tenascin-C regulates cell-matrix interaction in tendon. *TNC* contained a polymorphism in intron 17 where the number of GT dinucleotide repeats ranged from 3 to 21, and 95% of the alleles contained 12 to 17 GT repeats. Those subjects who were homozygous or heterozygous for the underrepresented alleles of *TNC* (containing either 13 or 17 GT repeats) were 6.2 times less likely to develop Achilles tendon injury [26] indicating that these variants of *TNC* may be protective from Achilles tendinopathy. *COL5A1* encodes the pro-a1 (V) chain of the type V collagen. Two variants of the *COL5A1* gene were associated with an increased risk of Achilles tendinopathy. In contrast, a third variant was underrepresented in subjects with Achilles tendinopathy and therefore was associated with a reduced likelihood of Achilles tendinopathy development [27]. A follow-up study published by the same research group from South Africa in collaboration with researchers from Australia replicated previous shown associations linking *COL5A1* gene variants to Achilles tendinopathy in both Australian and South African populations [33]. These studies demonstrate the relevance of the *TNC* and *COL5A1* gene polymorphisms in genetic predisposition to Achilles tendinopathy.

Other studies have assessed the contribution of haplotypes constructed from candidate polymorphisms, in an effort to find interactions between genes or gene products. A collaborative study investigated *COL27A1* gene polymorphisms in conjunction with *TNC*, as *COL27A1* is located in the same region of chromosome 9 as the *TNC* gene*.* Type XXVII collagen, encoded by *COL27A1*, is responsible for the key structural framework and tensile strength of the interstitial matrices. Although there were no significant associations between *COL27A1* and Achilles tendinopathy, a GCA haplotype (a set of genetic variants located on a single chromosome) constructed from one *COL27A1* polymorphism (rs946053) and two *TNC* polymorphisms (rs13321, rs2104772) showed significant association with Achilles tendinopathy [30]. The study of *COL5A1* and *MIR608* polymorphisms in South African and Australian cohorts found that a variant of *COL5A1* contains a putative polymorphic micro-RNA binding site. The results showed that polymorphisms rs71746744, rs16399 and rs1134170 in *COL5A1* and polymorphism rs4919510 in *MIR608*, which encodes a small micro-RNA, were all independently associated with Achilles tendinopathy, suggesting a role for these four variants on messenger ribonucleic acid (mRNA) stability and the resulting type V collagen synthesis [23]. A follow-up study of the same polymorphisms in a British cohort did not find any independent association between studied COL5A1, MIR608 or IL1β variants and Achilles tendinopathy as it was shown in Australian and South African cohorts [36]. However, an inferred allele combination constructed from *COL5A1* SNPs rs12722, rs3196378 and rs71746744 was associated with the risk of Achilles tendon pathology [36].

A further study conducted on South African subjects analysed polymorphisms in the gene encoding for matrix metalloproteinase 3 (MMP3) which is involved in the regulation of extracellular matrix homeostasis. Three of the investigated polymorphisms (rs679620, rs591058, rs650108) showed strong association with Achilles tendinopathy, and the most underrepresented haplotype in patients with Achilles tendinopathy indicated that this variant was protective against Achilles tendinopathy [31]. Moreover, since *MMP3* genotyping had been done on the same cohort as *COL5A1* reported by September (2009) [33], this study presented allelic combinations of *MMP3* rs679620 and *COL5A1* rs12722, which are associated with a lower risk of Achilles tendinopathy. Type V collagen is a substrate for MMP3; hence, genetic variants in *COL5A1* and *MMP3* genes could account for differences in the interactions between the proteins [31]. Further investigation of *MMP3* gene’s polymorphisms showed that an inferred haplotype of four SNPs (rs3025058, rs679620, rs591058 and rs650108) was associated with Achilles tendinopathy in an Australian cohort [37].

Polymorphisms in *COL12A1* (rs240736, rs970547) and *COL14A1* (rs4870723, rs1563392) were investigated as they both encode for proteins involved in the biological processes of fibrillogenesis and, like tenascin C, in the modulation of the tendon response to mechanical stress [34]. Additionally, one study of two cohorts from South Africa and Australia investigated polymorphisms in the three genes coding for type XI collagen that is homologous to type V collagen in function and structure [25]. Type XI collagen is usually expressed not only in cartilage but also in developing tendons. Several polymorphisms (*COL11A1* rs3753841 and rs1676486, *COL11A2* rs1799907) have been associated with lumbar disc herniation and rheumatoid arthritis. Whilst none of the polymorphisms were independently associated with Achilles tendinopathy, the construction of a pseudohaplotype consisting of three polymorphisms and the *COL5A1* polymorphism rs71746744 revealed a significant association with Achilles tendinopathy. It is hypothesized that the interaction of genes encoding for type V and XI collagens could modulate risk of Achilles tendinopathy, allowing for the possibility that the effects of type XI collagen variants in the developing tendon might affect the structural or functional properties of the mature tendon [25].

Genes involved in tendon homeostasis and particularly in the extracellular matrix (ECM) have also been investigated for their association with Achilles tendinopathy risk [24]. The ADAMTS (a disintegrin and metalloproteinase with thrombospondin motifs) family of proteinases are involved in ECM homeostasis and reported to be more highly expressed in pathologic than in healthy tendons. Tissue inhibitor of metalloproteinases (TIMP) inhibits the actions of MMPs and ADAMTS. Previously studied cohorts were genotyped for the *ADAMTS2* rs1054480, *ADAMTS5* rs226794, *ADAMTS14* rs4747096, *ADAMTS12* rs3740199 and *TIMP2* rs4789932 gene variants. Researchers found a significant association between the rs4789932 *TIMP2* variant and Achilles tendinopathy. The balance between TIMPs and MMPs could be a contributing factor for Achilles tendinopathy development [24]. The replication of the study of *MMP3* and *TIMP2* gene variants in a British cohort showed that a gene variant in *TIMP2* rs4789932 was associated with a reduced risk of Achilles tendon pathology in males [38]. The continued investigation of proteins involved in the tendon structure included research of fibrillin and elastin for their role in elasticity, strength and flexibility of tendons. The polymorphisms *FBN2* rs331079 and *ELN* rs2071307 were studied in Australian and South African cohorts, and the GG genotype in rs331079 was overrepresented in the group with Achilles tendinopathy, indicating an association between fibrillin and injury [35]. Another study conducted on the same cohorts investigated the contribution of genes encoding growth factors which play an important role in tendon growth and homeostasis. *TGFB1* and *GDF5* (encoding for transforming growth factor-β1 and growth/differentiation factor-5, respectively) were selected as candidates as these proteins had been shown to increase mechanical strength after gene transfection in Achilles tendon in experimentally injured animals [51]. This study showed a significant association of Achilles tendinopathy with *GDF5* rs143383. However, no association with *TGFB1* rs1800469 was identified [29].

A thorough analysis of polymorphisms in *COL5A1* identified this gene as one of the most likely predisposing factors for Achilles tendinopathy. However, several studies investigating polymorphisms in genes encoding for proteins interacting with type V collagen showed that it is important to be aware of possible connections and pathways whose interactions might be disrupted and therefore alter collagen structure and its functionality and lead to increased or decreased risk of Achilles tendinopathy.

### Genetic Contributors to the Pathways Involved in Apoptosis and Inflammation

Candidate genes involved in processes surrounding the development of Achilles tendinopathy, such as tendon turnover and inflammation, have also been considered as possible genetic risk factors. The presence of SNPs in cytokine genes, important contributors to the inflammatory response which have been shown to be upregulated in tendinopathy and mechanically loaded tendon were investigated. Interleukin-1β (IL1β) induces inflammatory mediators that upregulate the expression of proteins involved in degradation of the tendon extracellular matrix such as MMPs which target type V collagen. The IL1β receptor antagonist, IL1ra, is encoded by the *IL1RN* gene, and its variable number tandem repeat (VNTR) rs2234663 polymorphism has been previously associated with gastrointestinal diseases [52], osteoporotic fractures [53] and atherosclerosis [54]. Whilst genetic variants in *IL1β* (rs1143627 and rs16944) have been implicated to an increase in *IL1β* gene expression [55], interleukin-6 (IL6) was found to be linked to tenocyte apoptosis, which is a characteristic of tendinopathy. IL1β and IL6 may also affect *COL5A1* gene expression [32]. A SNP in *IL6* (rs1800795) was previously shown to alter *IL6* expression [56] which may lead to increased tenocyte apoptosis and therefore potentially increase risk for Achilles tendinopathy development. In total, the study investigated four polymorphisms in *IL1β*, *IL1RN* and *IL6*, although none of these polymorphisms were associated with the Achilles tendinopathy diagnosis in either of the South African and Australian population groups studied. However, inferred allele combinations constructed from previously studied *COL5A1* polymorphisms and these *IL1β, IL6* and *IL1RN* VNTR polymorphisms were associated with an increased risk of Achilles tendinopathy in combined groups [32]. This study concluded that genetic polymorphisms contributing to the changes in inflammatory pathways may be important contributors to the risk of Achilles tendinopathy.

Polymorphisms in genes encoding caspases (*CASP*) and nitric oxide synthases (*NOS*) have also been investigated as these molecules had been shown to be involved in pathways accompanying tendon cell apoptosis, and their expression has been found to be elevated in tendinopathy [28]. South African and Australian cohorts were genotyped for four polymorphisms (*CASP8* rs3834129, rs1045485, *NOS3* rs1799983 and *NOS2* rs2779249). A significant association between both *CASP8* polymorphisms (rs3834129, rs1045485) and Achilles tendinopathy was found in both populations. The D/D genotype of rs3834129 was associated with tendinopathy, whilst the C allele of rs1045485 was associated with the absence of Achilles tendinopathy. *NOS3* (rs1799983) and *NOS2* (rs2779249) were not associated with Achilles tendinopathy. However, the data presented in this study showed that the control group in the Australian cohort was not in Hardy-Weinberg equilibrium (HWE), which refers to constant proportions of allele and genotype frequencies in a population, and therefore, this association should be interpreted with caution. Deviations from HWE in the control group may indicate significant methodological flaws including selection bias, population stratification and genotyping errors [57]. Another drawback of this study was the conclusion that with an odds ratio (OR) of 1.67 for the presence of D/D genotype of rs3834129, the risk of Achilles tendinopathy was 68% higher in the tendinopathy group than in the control group [28]. An OR represents the odds that an outcome will occur given a particular exposure, compared to the odds of the outcome occurring in the absence of that exposure [58]. Therefore, OR does not represent the probability of the outcome, and risk assumptions in percentages may not be valid. Overall, this study was the first that investigated polymorphisms in caspase pathways.

Tumour necrosis factor receptor 1 gene *TNFRSF1A,* which signals inflammation and apoptosis in response to the tumour necrosis factor-alpha (TNFα), was investigated as a potential gene associated with Achilles tendinopathy [59]. *TNFRSF1A* rs4149577 had been associated with several musculoskeletal and inflammatory diseases; however, this study was the first to investigate this polymorphism in association with Achilles tendinopathy. Another polymorphism included in this study was the caspase-3 gene *CASP3* rs1049253, which was shown to influence *CASP3* mRNA expression. Caspase-3 is involved in cellular apoptosis, including roles in chromatin condensation and DNA fragmentation [60]. This study also investigated the influence of the copy number variant (CNV) spanning intron 11-intron 12 in *CASP8*. CNVs are segments of DNA greater than 1 kb in size that can influence phenotypes by changing gene dosage and disruption of coding sequences in DNA. The results did not show any significant association between Achilles tendinopathy, the investigated polymorphisms and CNV. This is possibly due to the study limitations, such as a relatively small sample size and a possible additional degree of error due to the rounding of copy number data into discrete calls, or may indicate that there is no association between these CNVs and Achilles tendinopathy [39].

While these studies indicate some links between polymorphisms in genes involved in apoptosis and inflammation and Achilles tendinopathy, the majority of these studies were unable to demonstrate that these genes are probable risk factors for Achilles tendinopathy. The processes of apoptosis and inflammation clearly play a role in the pathology of Achilles tendinopathy; however, further investigation should be undertaken to clarify the role of genetic contributors in this pathological processes.

## Conclusions

It is clear from previous research that biomechanical issues and training load errors are risk factors for Achilles tendinopathy [14]. This systematic review suggests that biomedical risk factors are an important consideration in the future study of propensity to the development of Achilles tendinopathy. Increased BMI and adverse lipid profile were associated with tendinopathy and may be important biomarkers of tendon pathology. It is evident that certain genetic markers contribute to the risk profile of Achilles tendinopathy; however, the demonstrated associations are currently somewhat ambiguous, and predictive power has not been demonstrated. Further investigation is required in this area. In addition, there appears to be diversity in the genes, dependent on geographical differences that are significantly associated with Achilles tendinopathy. This suggests that genetic risk factors for tendinopathy might be modified by geographic factors. It is evident that the risk of Achilles tendinopathy conferred by biomedical factors is complex and may be a result of the interplay between various genetic, biochemical and systemic factors which may be exacerbated by physical load. Further elucidation of biomedical risk factors will aid in the understanding of tendon pathology and patient risk, thereby informing prevention and management strategies for Achilles tendinopathy.

## Abbreviations

ADAMTS: A disintegrin and metalloproteinase with thrombospondin motifs
*CASP*: Gene encoding caspases
CNV: Copy number variant
*COL11A1*: Gene encoding pro-alpha1(XI) chain of type XI collagen
*COL11A2*: Gene encoding pro-alpha2(XI) chain of type XI collagen
*COL12A1*: Gene encoding alpha chain (XI) of type XII collagen
*COL14A1*: Gene encoding alpha chain (XI) of type XIV collagen
*COL27A1*: Gene encoding Type XXVII collagen
*COL5A1*: Gene encoding pro-alpha1 (V) chain of the type V collagen
DNA: Deoxyribonucleic acid
ECM: Extracellular matrix
*ELN*: Gene encoding elastin
*FBN2*: Gene encoding fibrillin-2
*GDF5*: Gene encoding growth/differentiation factor-5
HDL-C: High-density lipoprotein cholesterol
HWE: Hardy-Weinberg equilibrium
*IL1RN*: Gene encoding IL1β receptor antagonist IL-1ra
IL1β: Gene encoding Interleukin-1β
*IL6*: Gene encoding Interleukin-6
*MIR608*: Gene encoding small micro-RNA
*MMP3*: Gene encoding matrix metalloproteinase 3
mRNA: Messenger ribonucleic acid
NOS: Newcastle-Ottawa Quality Assessment Scale
*NOS*: Gene encoding nitric oxide synthases
OCEBM: Oxford Centre for Evidence-based Medicine levels of evidence
OR: Odds ratio
PICO: Patient Intervention, Comparison, Outcome
PRISMA: Preferred Reporting Items for Systematic Reviews and Meta-Analyses
SNP: Single nucleotide polymorphism
TG: Triglycerides
*TGFB1*: Gene encoding transforming growth factor-β1
TIMP: Tissue inhibitor of metalloproteinases
*TNC*: Gene encoding tenascin-C
*TNFRSF1A*: Tumour necrosis factor receptor 1 gene
TNFα: Tumour necrosis factor-alpha
VISA-A questionnaire: Victorian Institute of Sport Assessment-Achilles questionnaire
VNTR: Variable number tandem repeat
